# Enhanced gastrocnemius-mimicking lower limb powered exoskeleton robot

**DOI:** 10.1186/s12984-025-01703-y

**Published:** 2025-08-04

**Authors:** Tianchi Chen, Zhi Liu, Chaoyang Li, Xiaoan Chen, Jianjun Hu, Ye He

**Affiliations:** 1 State Key Laboratory of Mechanical Transmission for Advanced Equipment, Chongqing, 400044 China; 2 College of Mechanical and Vehicle Engineering, Chongqing University, Chongqing, 400044 China; 3 College of Intelligent Engineering, Chongqing City Management College, Chongqing, 401331 China

**Keywords:** Bionic device, Gastrocnemius-mimicking, Exoskeleton robot, Biarticular muscles, Wearable technology

## Abstract

**Background:**

Lower limb muscle bionic devices have attracted significant attention in rehabilitation and assistive sports technology. Despite advancements in mimicking human movement, current devices still face challenges in enhancing strength and movement capabilities. These devices often focus on monoarticular muscles, overlooking the synergistic effects of biarticular muscles and their role in energy transfer, which limits the overall improvement in movement performance.

**Methods:**

This study presents an enhanced gastrocnemius-mimicking exoskeleton robot (EGME), leveraging the biarticular characteristics of the muscle. The device delivers force spanning both the knee and ankle joints to provide vertical support and forward propulsion in an underactuated manner during locomotion. Its effectiveness was evaluated through experimental trials involving five volunteers performing level walking and squat holding tasks.

**Results:**

Experimental results showed that the EGME significantly reduced gastrocnemius activation, improved exercise endurance, and enhanced ankle stability. Activation decreased by up to 46.4% during walking and by an average of 59.8% during the short-duration squat holding task, while endurance time in the long-duration squat holding task increased by a factor of 7.79 with the exoskeleton.

**Conclusion:**

This study demonstrates the strong potential of biarticular exoskeletons to enhance muscle function and movement performance, offering new insights into bionic device design. These findings suggest broad applicability in performance enhancement and rehabilitation. Future research should further explore their effects on inter-joint coordination and kinematic coupling to refine the design and functionality of such systems.

## Background

In recent years, lower limb musculoskeletal bionic devices have attracted widespread attention in the fields of rehabilitation medicine and assistive movement technology, offering effective support for functional recovery and daily activities [1–5]. These devices mimic the physiological characteristics of the human body, offering support to rehabilitation patients and facilitating the restoration of muscle strength and coordination. Additionally, they exhibit significant potential in enhancing movement and improving physical fitness [6–9].

While notable progress has been made in mimicking human lower limb movements, most current bionic devices primarily focus on enhancing the strength of monoarticular muscles [10–12]. These devices typically assist in rehabilitation or improve movement performance by replicating the functions of specific muscle groups. However, human lower limb movements often involve complex coordination across multiple joints, and relying solely on the enhancement of monoarticular muscles makes it challenging to improve overall movement capabilities comprehensively [13–15]. Furthermore, existing devices generally overlook the synergistic roles of biarticular muscles (such as the gastrocnemius) in movement. Biarticular muscles span two joints and play a critical role in human locomotion by coordinating actions across multiple limb segments, thereby optimizing movement efficiency and reducing energy waste [16]. However, traditional bionic devices lack adequate consideration of biarticular muscle functions, resulting in limited effectiveness in mimicking natural movements [17–21].

Another challenge in current lower limb bionic devices is the lack of efficient energy transmission [22–24]. In natural movement, biarticular muscles work in concert to transfer force and energy between joints, resulting in smoother, more energy-efficient motion patterns. For instance, in mid-single-leg stance, although soleus (SOL) and gastrocnemius (GAS) muscles are isometric, their forces transfer energy between the leg and trunk in opposite directions, ensuring support and forward propulsion. Their combined reaction forces enable efficient energy exchange between the trunk and leg, maintaining the energy state of both segments while supporting ballistic body motion [25]. By focusing solely on monoarticular muscles, existing devices are unable to optimize energy transfer, thus hindering improvements in movement performance [26, 27]. Furthermore, the absence of effective energy transfer mechanisms leads to unnecessary energy consumption, reducing the endurance of the device wearer [28, 29].

While many bionic devices offering joint torque assistance have demonstrated effectiveness in the short term [30–34], prolonged use can cause discomfort due to misalignment between the exoskeleton and the human joint rotation centers, limiting their practical application [35, 36]. Furthermore, excessive reliance on monoarticular assistance during long-term rehabilitation can lead to uneven joint loading and increased fatigue.

The GAS is a biarticular muscle that spans both the knee and ankle joints. It is particularly prominent in storing and releasing energy during the gait cycle, thereby significantly improving movement efficiency and stability [37–39]. However, existing gastrocnemius-mimicking devices mostly adopt passive or quasi-passive mechanisms to enhance balance ability [40] and improve prosthetic performance [41, 42]. The research and simulation of GAS remain limited, making it difficult to fully leverage its potential in underactuation and energy transfer [43–45].

To address the above challenges, this work develops an enhanced gastrocnemius-mimicking lower limb powered exoskeleton robot (EGME) on the basis of an in-depth analysis of the biomechanical characteristics of the GAS. This device provides biarticular energy support parallel to the GAS between the knee and ankle joints, significantly reducing the activation intensity of the GAS during the walking gait cycle and enhancing movement endurance. Experimental validation with five volunteers demonstrated that the device exhibited excellent performance in tasks such as level walking and squat holding, with the GAS activation intensity reduced by 46.4% and 59.8%, respectively, and the endurance time during prolonged squat holding increased by 7.79 times. These results indicate that the EGME has significant potential for enhancing muscle function and optimizing movement performance. Furthermore, this study provides new insights for future exoskeleton designs, advancing bionic technology in the fields of athletic performance enhancement and rehabilitation.

## Methods

### Biomechanical analysis

#### Kinematics and kinetics

**Fig. 1:**
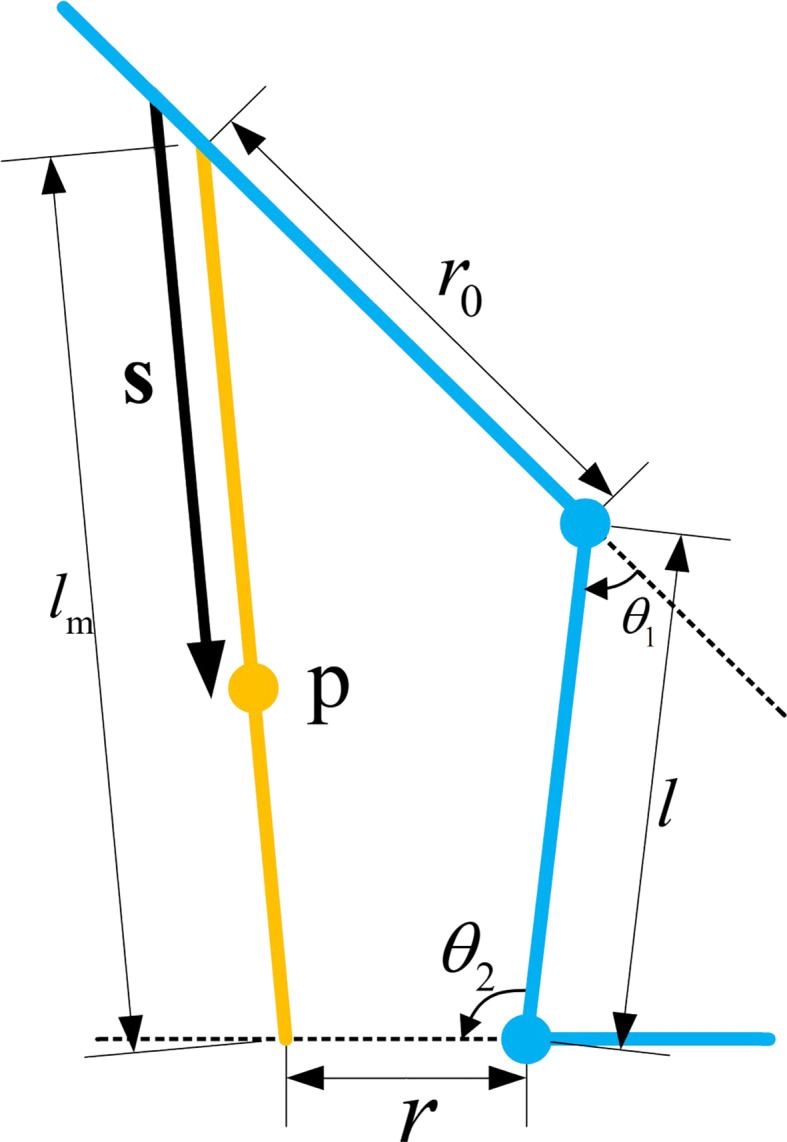
Sketch of the kinematics of the EGME. The blue color represents the human lower limb segments. The orange color represents the EGME

A kinematic sketch of the EGME is shown in Fig. 1. The coordinates of a material point $\:p$ on the muscle are:

1 $$\:\varvec{p}=\left(\begin{array}{c}0\\\:{r}_{0}\end{array}\right)\left(1-s\right)+\left(\begin{array}{c}l\text{cos}{\theta\:}_{1}+r{cos}({\theta\:}_{1}+{\theta\:}_{2})\\\:l\text{sin}{\theta\:}_{1}+r{sin}({\theta\:}_{1}+{\theta\:}_{2})\end{array}\right)s$$

When the knee angle $\:{\theta\:}_{1}=0$and the ankle angle $\:{\theta\:}_{2}=\frac{\pi\:}{2}$, the coordinates of the material point $\:{p}^{L}$are:

2 $$\:{\varvec{p}}^{L}=\left(\begin{array}{c}-{r}_{0}\text{sin}{\theta\:}_{1}\\\:{r}_{0}\text{cos}{\theta\:}_{1}\end{array}\right)\left(1-s\right)+\left(\begin{array}{c}l\text{cos}{\theta\:}_{1}-r\text{sin}{\theta\:}_{1}\\\:l\text{sin}{\theta\:}_{1}+r\text{cos}{\theta\:}_{1}\end{array}\right)s$$

At this point, the total length of the EGME:

3 $$\:{l}_{m}=\sqrt{{k}^{2}+{r}_{0}^{2}+2{r}_{0}k{cos}\left({\theta\:}_{1}+\frac{r{\theta\:}_{2}}{k}\right)}$$

where $$\:k=\sqrt{{r}^{2}+{l}^{2}-2rl{cos}{\theta\:}_{2}}$$

**Fig. 2:**
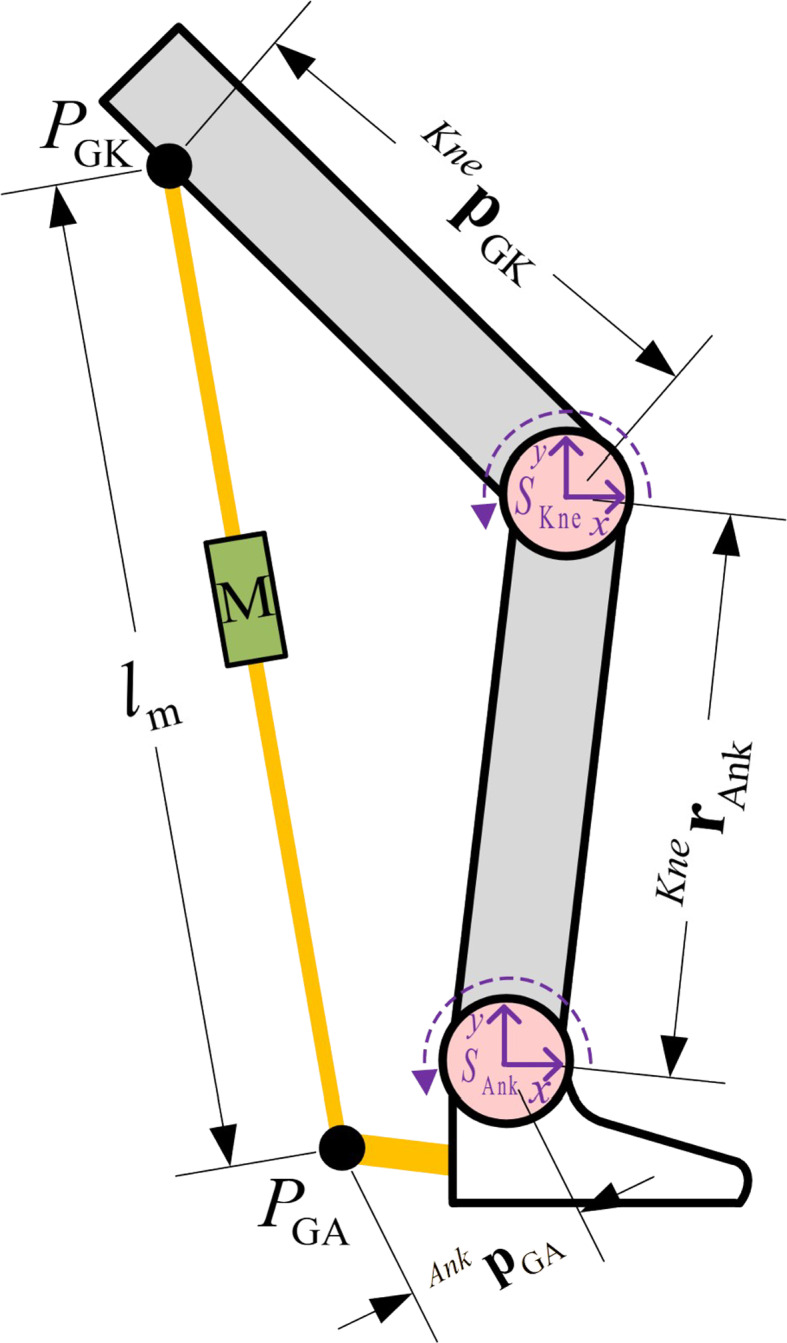
Schematic of the kinetics of the EGME

To facilitate kinetic representation, we replace each parameter in the sketch in Fig. 1 with a common form for kinetic calculations, as shown in Fig. 2.

As shown in Fig. 2, the EGME is depicted via the virtual work and virtual displacement method, with both the knee and ankle reference frames located on the articular axis. 

**Table 1:** Definitions for kinetic description

Symbols	definitions	descriptions
$\:{{}^{\text{Kne}}\varvec{T}}_{\text{Ank}}({q}_{k},{q}_{a})$	$$\:=\hspace{0.33em}\left[\begin{array}{cc}\text{Ro}{\text{t}}_{z}({q}_{k}+{q}_{a})&\:\text{Ro}{\text{t}}_{z}\left({q}_{k}\right){{}^{\text{Kne}}\varvec{r}}_{\text{Ank}}\\\:0&\:1\end{array}\right]$$	Geometric transformation matrix from knee to ankle joint.
$\:{{}^{\text{Ank}}\varvec{T}}_{\text{Kne}}({q}_{a},{q}_{k})$	$$\:=\hspace{0.33em}\left[\begin{array}{cc}\text{Ro}{\text{t}}_{z}({q}_{k}+{q}_{a}{)}^{T}&\:\text{-Ro}{\text{t}}_{z}({q}_{a}{)}^{T}{{}^{\text{Kne}}\varvec{r}}_{\text{Ank}}\\\:0&\:1\end{array}\right]$$	Geometric transformation matrix from ankle to knee joint.
$\:{{}^{\text{Kne}}\varvec{p}}_{\text{GK}}$	The position vector of the EGME connected to the fixed point $\:{P}_{\text{GK}}$ at the knee joint relative to the origin of $\:{S}_{\text{kne}}$ in the knee reference frame.	
$\:{{}^{\text{Ank}}\varvec{p}}_{\text{GA}}$	The position vector of the EGME connected to the fixed point $\:{P}_{\text{GA}}$ at the ankle joint relative to the origin of $\:{S}_{\text{Ank}}$ in the ankle reference frame.	
$\:{}^{\text{Kne}}\varvec{l}({q}_{k},{q}_{a})$	$\:=\hspace{0.33em}{{}^{\text{Kne}}\varvec{p}}_{\text{GA}}({q}_{k},{q}_{a})-{{}^{\text{Kne}}\varvec{p}}_{\text{GK}}$	Length vector of the EGME at the knee joint.
$\:{}^{\text{Ank}}\varvec{l}({q}_{a},{q}_{k})$	$\:=\hspace{0.33em}{{}^{\text{Ank}}\varvec{p}}_{\text{GK}}({q}_{a},{q}_{k})-{{}^{\text{Ank}}\varvec{p}}_{\text{GA}}$	Length vector of the EGME at the ankle joint.

According to Table 1, by defining the relative directions and positions of the reference frames $\:{S}_{\text{kne}}$and$\:{S}_{\text{Ank}}$, the fixed points $\:{P}_{\text{GA}}$ and $\:{P}_{\text{GK}}$ of the EGME on the thigh and shank can be expressed as:

4 $$\begin{gathered}^{{\text{Kne}}}{p_{{\text{GA}}}}({q_k},{q_a}){ = ^{{\text{Kne}}}}{T_{{\text{Ank}}}}{({q_k},{q_a})^{{\text{Ank}}}}{p_{{\text{GA}}}} \hfill \\^{{\text{Ank}}}{p_{{\text{GK}}}}({q_a},{q_k}){ = ^{{\text{Ank}}}}{T_{{\text{Kne}}}}{({q_a},{q_k})^{{\text{Kne}}}}{p_{{\text{GK}}}} \hfill \\ \end{gathered} $$

where $\:{q}_{k}$ and $\:{q}_{a}$ represent the angles of the knee and ankle, respectively. The torque generated by the EGME at the knee and ankle joints can be obtained via the cross product:

5 $$\begin{gathered}^{{\text{Kne}}}\tau \:({q_k},{q_a}){ = ^{{\text{Kne}}}}{p_{{\text{GK}}}} \times \:{F_{GME}}({q_k},{q_a}) \hfill \\^{{\text{Ank}}}\tau \:({q_a},{q_k}){ = ^{{\text{Ank}}}}{p_{{\text{GA}}}} \times \:{F_{{\text{GME}}}}({q_a},{q_k}) \hfill \\ \end{gathered} $$

where $\:{F}_{GME}({q}_{k},{q}_{a})$ and $\:{F}_{\text{GME}}({q}_{a},{q}_{k})$ represent the forces generated by the EGME at the knee and ankle joints, respectively, and are dependent on the active control of the system algorithm. Since $\:{{}^{\text{Kne}}\varvec{p}}_{\text{GK}}$ and $\:{{}^{\text{Ank}}\varvec{p}}_{\text{GA}}$ are interchangeable with the corresponding moment arm vectors, the torques generated by the EGME at the knee joint and ankle joint are as follows:

6 $$\begin{gathered}\:\tau {\:_{{\text{GAS,Kne}}}}({q_k},{q_a}){ = ^{{\text{Kne}}}}\tau \:({q_k},{q_a}) \cdot \:{e_Z} \hfill \\\tau {\:_{{\text{GAS,Ank}}}}({q_a},{q_k}){ = ^{{\text{Ank}}}}\tau \:({q_a},{q_k}) \cdot \:{e_Z} \hfill \\ \end{gathered} $$

#### Objectives of the EGME function

**Fig. 3:**
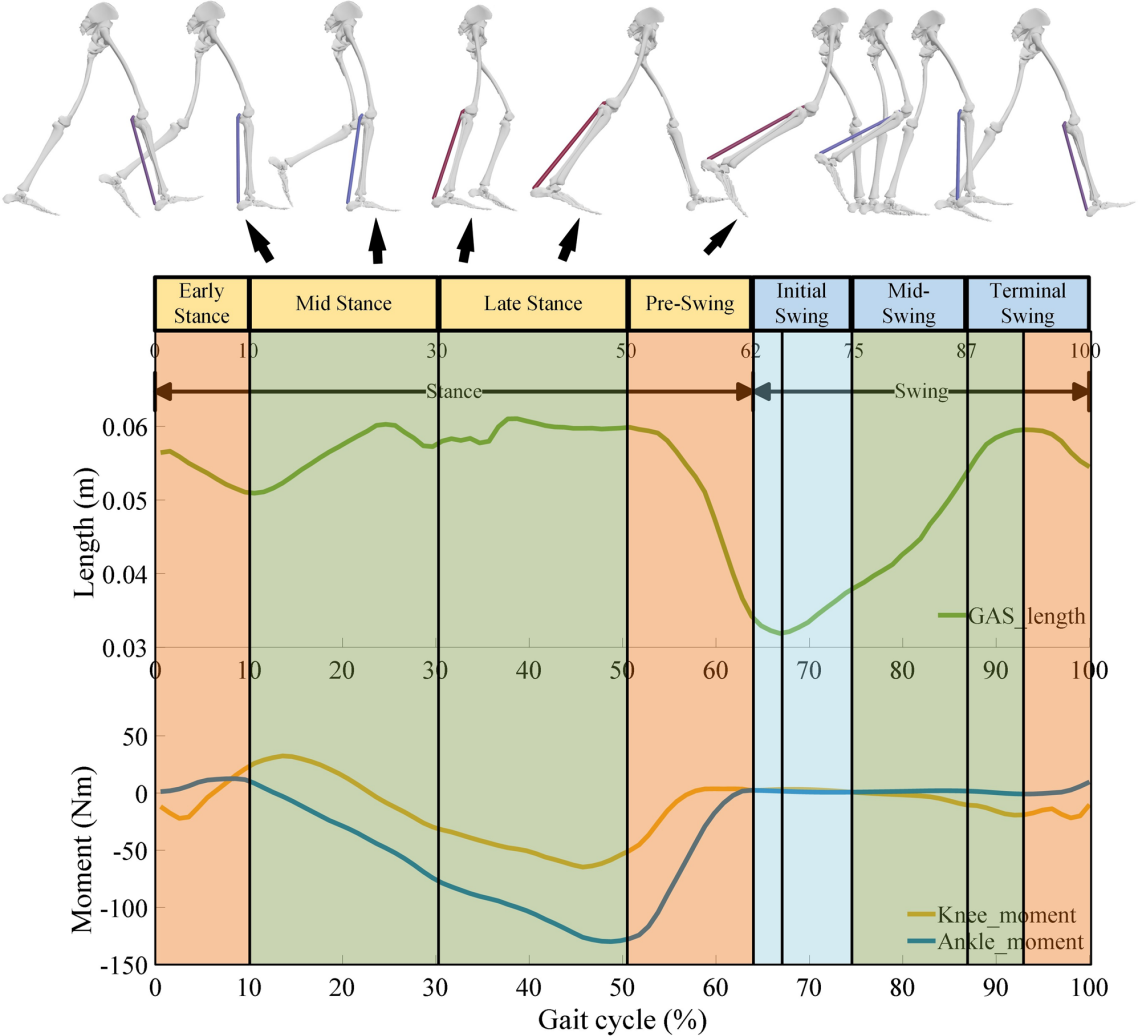
Division of gait cycle phases and corresponding changes in gastrocnemius fiber length and knee-ankle joint moments. The green curve represents the change in GAS muscle fiber length. The orange and blue curves represent the changes in knee and ankle joint moments, respectively. In the gait phases with a red background, the EGME primarily generates thrust; in the gait phases with a green background, the EGME primarily generates tensile force; in the gait phases with a blue background, the EGME outputting zero force based solely on the position change calculated by Eq. (3). The black arrows indicate the direction of the ground reaction force

As illustrated in Fig. 3, the GAS engages in distinct functional roles across different phases of the gait cycle, leading to varying activation levels. These phase-dependent activation patterns provide important insights for the control strategy of the EGME.

During the early single-leg stance phase (10–25% of the gait cycle), the GAS undergoes eccentric contraction, gradually increasing vertical support for the trunk while slowing down its forward movement. The net effect is an absorption of the trunk’s energy [25]. According to the torque requirements of the knee and ankle joints, the EGME should apply an upward thrust to the femur with a forward horizontal component, thus providing positive energy output in both the vertical and horizontal directions for the trunk. Based on the kinematic characteristics of the gastrocnemius, the muscle is stretched during this phase [46], and the EGME uses the results from Eq. (3) as the target for position tracking to achieve extension.

In the mid-stance phase of single-leg support (approximately 30% of the gait cycle), the GAS approaches isometric contraction and slightly resists forward movement of the trunk. The GAS transfers energy from the trunk to the leg, ensuring support and forward propulsion for both the leg and the trunk through this energy transfer, which is opposite in direction to the SOL [25, 47]. During this phase, the EGME provides thrust under near-isometric conditions, transferring energy from the motor to the leg and simultaneously providing support and forward propulsion.

In the late single-leg stance phase, the GAS transfers energy to the leg to assist in forward propulsion. During this stage, the GAS fibers are in an isometric state and gradually shorten. The EGME adopts the same thrust pattern as in the mid-stance phase of single-leg support. In the pre-swing phase, the GAS plays a key role in initiating the swing [48]. Due to the delayed deactivation of the muscle, GAS activation ceases in the mid-pre-swing phase, with force output gradually diminishing until the end of pre-swing. The GAS transfers nearly all its energy to the leg to assist with the start of the swing [25]. During this phase, the EGME rapidly shortens and applies tensile force, with power and energy output to the leg maintained throughout the entire pre-swing phase.

### Control scheme

**Fig. 4:**
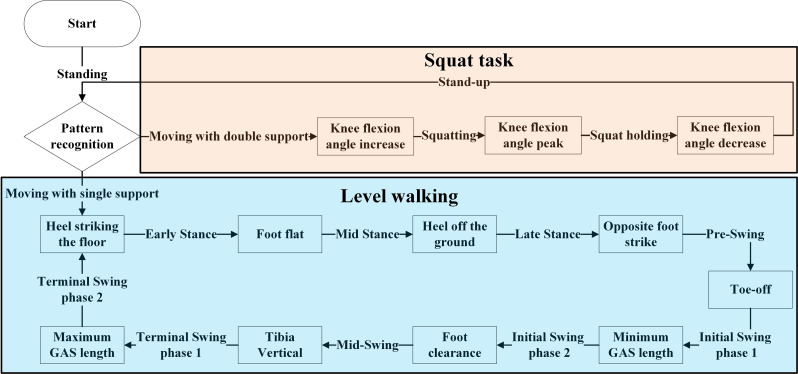
Finite state machine of the EGME

As shown in Fig. 4, to effectively distinguish the different phases in the tasks of level walking and squat task, this study employs a finite state machine (FSM). By defining distinct states and transition rules between states, the FSM accurately captures the dynamic changes from one phase to another within the gait cycle.

**Fig. 5:**
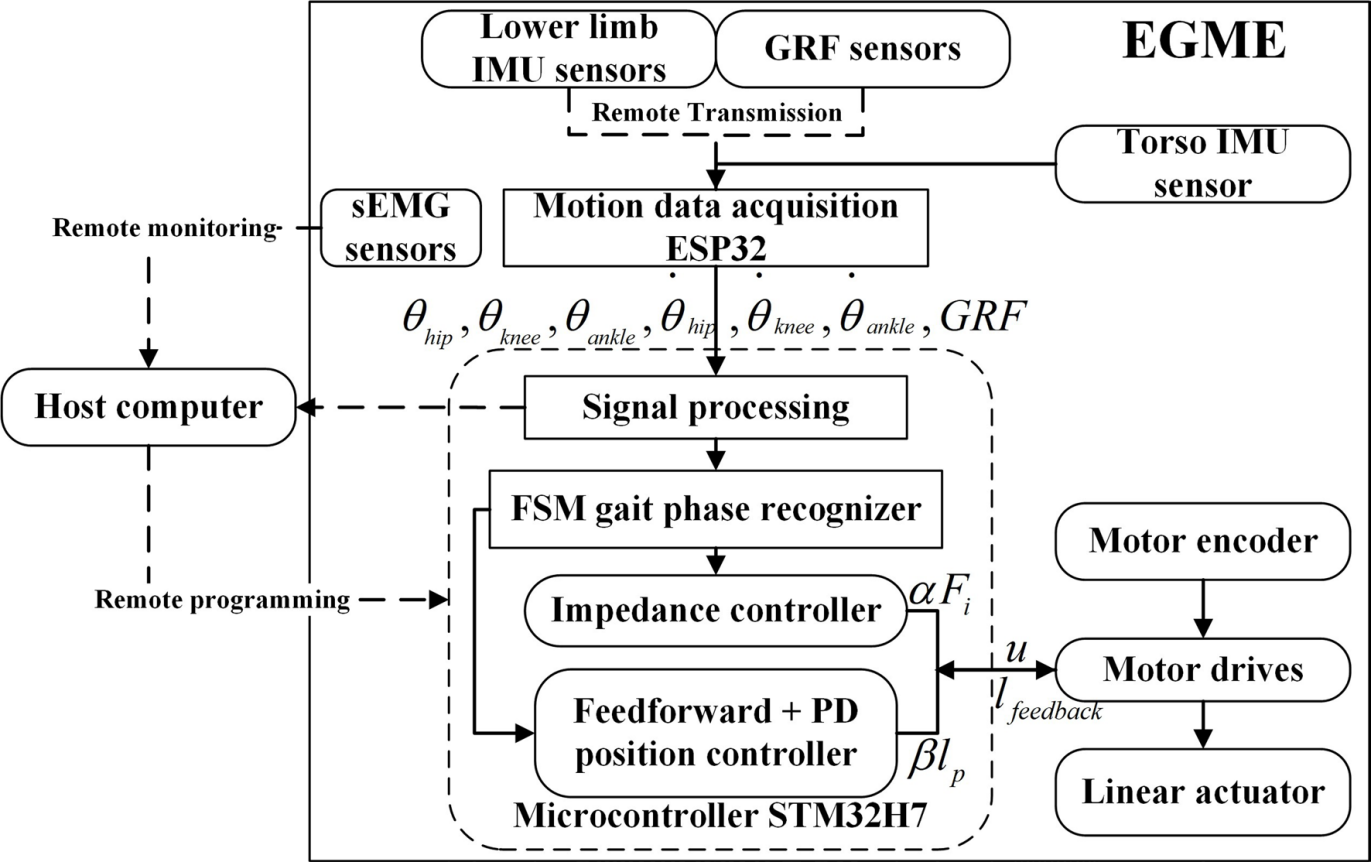
Control structure of the EGME

Considering the distinct characteristics exhibited by the GAS during different gait phases (such as isometric contraction and quasi-isotonic contraction), this study employs a force-position parallel control strategy to ensure that the EGME provides adequate support force while adapting to human movement. This strategy combines impedance control with feedforward and PD-based position control and dynamically adjusts the weighting of the two based on gait phase recognition through the FSM, achieving compliant and stable exoskeleton control. As shown in Fig. 5, the control strategy in this study consists of two components: force control and position control, with the weighting of each component allocated based on the gait phase:

7 $$\:u=\alpha\:{F}_{i}+\beta\:{l}_{p}$$

8 $$\:{F}_{i}={K}_{s}\varDelta\:l+{D}_{s}\frac{d}{dt}\varDelta\:l$$

9 $$\:{l}_{p}={l}_{m}+{K}_{p}\varDelta\:l+{D}_{p}\frac{d}{dt}\varDelta\:l$$

Where $\:{F}_{i}$ represents the output of impedance control force, $\:{l}_{p}$ is the position control signal, and $\:\alpha\:,\beta\:$ is the dynamically adjusted weighting factor. $\:{K}_{s}$ is the stiffness coefficient, with $\:{K}_{s}={K}_{s0}+\gamma\:{F}_{ref}$ during the stance phase and $\:{K}_{s}=0.05{K}_{s0}$ during the swing phase. $\:{D}_{s}$ is the damping coefficient, with $\:{D}_{s}={D}_{s0}+\eta\:\left|{v}_{feedback}\right|$ during the stance phase and $\:{D}_{s}=0.25{D}_{s0}$ during the swing phase. $\:\varDelta\:l={l}_{m}-{l}_{feedback}$. $\:{K}_{p}$ is the proportional gain, and $\:{D}_{p}$ is the derivative gain. During the isometric contraction phase, $\:\alpha\:\gg\:\beta\:$, while during the Quasi-Isotonic phase, $\:\alpha\:\ll\:\beta\:$. Weight adjustment is carried out using a sigmoid transition function:

10 $$\:\alpha\:=\frac{1}{1+{e}^{-\lambda\:\left(t-{t}_{c}\right)}},\hspace{0.33em}\beta\:=1-\alpha\:$$

where $\:{t}_{c}$ is the gait phase transition timing, and $\:\lambda\:$ controls the transition speed.

In the squat task, the EGME primarily assists by supporting body weight and maintaining joint stability. To enhance the EGME’s effectiveness during squat task, an adaptive support force $\:{F}_{sq}$ is introduced into the force-position parallel control strategy. This force dynamically adjusts to compensate for variations in load distribution, reducing muscular strain and improving stability during squat-hold.

The adaptive support force $\:{F}_{sq}$ is influenced by multiple factors, including the user’s body mass, gravitational force, vertical acceleration, and the exoskeleton’s inclination. The user’s body mass $\:M$ is estimated through onboard sensors or predefined values. The vertical acceleration of the center of mass, denoted as $\:{a}_{h}$, is computed using IMU data, capturing changes in movement dynamics. The inclination angle $\:\gamma\:=\frac{\pi\:}{2}+{\theta\:}_{1}-{\theta\:}_{2}-{arccos}\frac{{r}_{0}^{2}+{l}_{m}^{2}-{r}^{2}-{l}^{2}+2rl{cos}{\theta\:}_{2}}{2{r}_{0}{l}_{m}}$ represents the actuator’s deviation from the vertical axis. The adaptive support force is formulated as:

11 $$\:{F}_{sq}=\left(1-{e}^{-\lambda\:t}\right)\frac{M\left(g+{a}_{h}\right)}{{cos}\gamma\:}$$

During the squatting phase, the $\:{F}_{sq}$ gradually increases, compensating for the additional braking load on the muscles. In the squat holding phase (isometric phase), where the user maintains a static posture with a fixed knee angle, the EGME offers stable support to reduce sustained muscle exertion. Here, $\:{F}_{sq}$ stabilizes, allowing the actuator to bear part of the user’s body weight, thereby minimizing metabolic cost. During the stand-up phase, as the center of mass rises and the knee extends, the EGME provides an assistive force to reduce muscular demand. At this stage, $\:{F}_{sq}$ gradually decreases to prevent abrupt force variations that could cause discomfort or instability. $\:{F}_{sq}$ is integrated into the force-position parallel control framework, modifying the $\:u$ to adjust across different motion phases:

12 $$\:u=\alpha\:\left({F}_{i}+{F}_{sq}\right)+\beta\:{l}_{p}$$

### Simulation analysis

To evaluate the impact of EGME on lower limb movement, this study conducted a human-machine interaction analysis using the OpenSim 4.5 simulation platform with the Gait10DOF18Musc model. This model, which includes the trunk, pelvis, and legs, features 10 degrees of freedom and 18 musculotendon units, serving as a simplified representation of the lower limbs. To systematically compare the effects of EGME and monoarticular exoskeletons on human movement, both an ankle exoskeleton and an EGME exoskeleton were incorporated into the model.

The monoarticular exoskeleton model was developed to assist ankle movement by incorporating a torsional spring at the ankle joint, implemented using the CoordinateLimitForce element. This force element is typically utilized to restrict the range of motion by activating a spring-damper system when a predefined limit is exceeded. Specifically, the CoordinateLimitForce was configured to generate a plantarflexion moment when the dorsiflexion angle surpasses 5 degrees, with an upper stiffness coefficient of 10 N·m/degree. To prevent excessive plantarflexion, a lower stiffness coefficient of 1 N·m/degree was assigned, with a lower limit set at -90 degrees, allowing a full range of plantarflexion motion. A small damping coefficient of 0.01 N·m·s/degree was introduced to refine the force transition.

A bidirectional translational actuator system was implemented to simulate the function of the EGME. This system consists of two PathActuator components, responsible for generating push and pull forces along a defined path between the femur and the calcaneus.

### Implementation of the EGME system

**Fig. 6:**
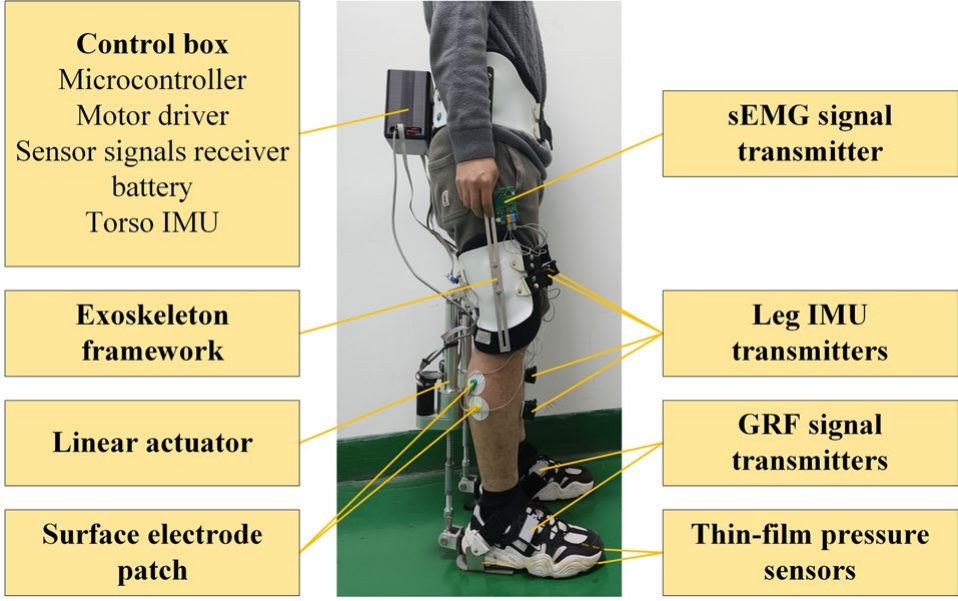
Overall structure of the EGME

As illustrated in Fig. 6, the device integrates a lower limb anthropomorphic framework on the basis of anthropometric data with non-anthropomorphic driver modules connected to the thighs and heels. The driver modules incorporate the calf muscle simulation mechanism designed in this study, which is capable of simulating and enhancing the gastrocnemius muscles of adults with heights ranging from 165 to 185 cm. The exoskeleton has a mass of 8.0 kg (excluding the battery) and a maximum structural load capacity of 120 kg, which includes the wearer, the exoskeleton itself, and any additional external load. This capacity refers to the total weight the exoskeleton is designed to support while maintaining structural integrity and effective load transmission. The weight is transmitted to the ground through the connection points between the exoskeleton and the wearer’s heels, thereby reducing the pressure exerted by the exoskeleton and the load on the wearer’s skeletal structure.

The driver modules consisted of a motor, synchronous belt, and ball screw assembly, specified as follows: frameless direct-drive high-torque miniature DC motors (RE 40, graphite brushes, 150 W, Maxon, Switzerland), incremental encoders (HEDL 5540, 500 CPT, 3 channels, with RS 422 line drivers, AVAGO, USA), timing belts with a transmission ratio of 2:1, and custom-designed electric cylinders capable of providing an extension range of 0–200 mm to accommodate variations in leg length due to different heights and movements. The control system utilizes a real-time embedded microcontroller (STM32F446RCT6, STMicroelectronics) for sensor signal acquisition, posture detection, motion intention prediction, and motor control. Communication with the motor drivers is achieved via a CAN bus at a speed of 1 Mbit/s, with encoder measurements relayed back to the motor drivers. The control system, motor drivers, and battery are integrated into a backpack or a small box that is worn on the back for portability.

The system accommodates five degrees of freedom (DOFs), including both active and passive DOFs, to align with human biomechanics and ensure natural leg movements. These DOFs include hip flexion/extension (0°–90°), hip adduction/abduction (− 45°–45°), hip internal/external rotation, knee flexion/extension (0°–90°), and ankle dorsiflexion/plantarflexion (− 45°–45°). While Figs. 1 and 2 primarily illustrate the DOFs engaged during sagittal plane motion, the exoskeleton design also incorporates passive DOFs to avoid excessive constraints on natural movement. To ensure wearer safety, the range of motion for each joint is software-limited to slightly below the normal physiological range. The exoskeleton system seamlessly integrates all functionalities, including signal acquisition and analysis, human intent recognition and torque calculation, and actuator control, thereby eliminating the necessity for external support devices.

### Experimental protocol

Five healthy adult male volunteers, ranging in age from 23 to 30 years, participated in this study. The participants presented a mean height of 176 cm (± 11 cm) and a mean weight of 67.8 kg (± 6.2 kg). None of the participants had any prior history of orthopedic, muscular, or neurological disorders. All procedures were approved by the Ethics Committee of Chongqing University, and written informed consent was obtained from each participant. Prior to the experiment, all participants were informed of the study’s objectives and procedures.

To facilitate real-time monitoring of both the exoskeleton and the human body, inertial measurement units (IMUs) were strategically placed on the torso, thighs, shanks and feet to continuously capture hip, knee and ankle joint angles. The IMUs used in the study included custom-designed boards integrating a signal acquisition module (JY61P, Witmotion, China) and a signal transmission module (ESP32-C3, Espressif Systems, China) for the thigh, shank, and foot. Additionally, an onboard BMI088 IMU (Bosch Sensortec GmbH, Germany) was used for torso measurement. Plantar pressure was measured using two thin-film pressure sensors (RX-D4046, Crownto, China) positioned at the toe and heel. The collected data were employed to identify distinct periods of the stance and swing phases.

Surface electromyography (sEMG) sensors were affixed to the GAS to measure muscle electrical activity, with a sampling frequency of 1000 Hz. The sEMG signals were recorded using a muscle electrical sensor kit (Sichiray, China). Upon acquisition, the EMG signals were first processed to remove the direct current (DC) offset, thereby resetting the signal baseline to zero. The signals were subsequently subjected to a dual-pass second-order Butterworth bandpass filter with a frequency range of 20–450 Hz. Following filtering, full-wave rectification was performed to convert all negative signal components to positive values. To generate a smooth linear envelope of the sEMG signals, a second-order low-pass Butterworth filter with a cutoff frequency of 6 Hz was applied.

Prior to the sEMG measurements conducted during the experiments, participants performed maximum voluntary contraction (MVC) tasks, following a protocol adapted from [49]. These tasks were essential for normalizing the amplitude of the sEMG signals and ensuring data comparability. The MVC measurement was conducted with participants in a standing position, keeping the knee and hip extended. They were instructed to perform an MVC of the GAS through ankle plantarflexion, sustaining the contraction for 5 s. This was repeated three times with a 3-minute rest period to prevent fatigue. sEMG recorded muscle activity, and the highest recorded value across the trials was taken as the MVC. In the postprocessing phase, the filtered sEMG signals were normalized based on the peak activity levels obtained from the MVC tests. The normalized signals were then averaged to evaluate the relative muscle activity levels. A one-way ANOVA was conducted to assess the differences in mean and peak gastrocnemius activation among conditions (FREE, ASSIST, TRANSPARENT). Post-hoc Tukey’s HSD tests were performed to determine pairwise differences between conditions when significant main effects were observed.

**Fig. 7:**
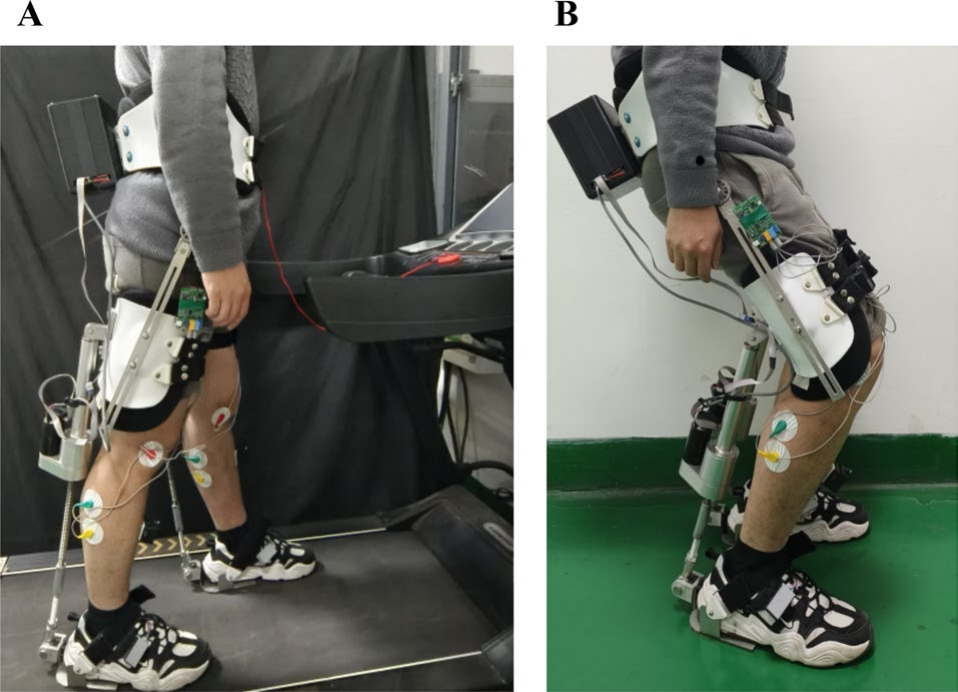
Scenarios of the device validation experiments. (**A**) Level walking. (**B**) Squat task

#### Level walking experiment

Prior to donning the EGME, participants adopted a natural upright standing posture. The anthropomorphic framework and the initial position of the EGME were adjusted according to each participant’s height. Each participant was given thirty minutes to familiarize themselves with walking while wearing the exoskeleton and to receive adequate rest before the formal experiments commenced. As depicted in Fig. 7A, subjects walked naturally on a treadmill at a predetermined speed under three distinct conditions, with each condition being repeated four times. A thirty-minute rest interval was provided between each experimental condition, and each walking trial lasted five minutes. The specific experimental conditions were as follows:

1. Free mode (FREE): Participants wore a gait and physiological information acquisition system integrated with IMUs, plantar pressure sensors, and electromyography sensors. They walked at a natural speed to collect gait parameters and activation intensity of the gastrocnemius muscles during movement.
2. Transparent mode (TRANSPARENT): Participants walked while wearing the EGME, which was set to a zero-assist mode. This condition was designed to assess the impact of the EGME on the participant’s walking patterns without providing any assistance.
3. Assist mode (ASSIST): Participants walked with the EGME actively augmenting the GAS. This condition aimed to evaluate the extent and characteristics of the assistance provided by the EGME.

Heel strike events were utilized to segment the gait data, marking the beginning and end of each gait cycle. The duration between heel strikes was normalized to the percentage of the gait cycle to ensure consistency in the data analysis.

#### Squat task experiment

Initially, participants performed a short-duration squat holding experiment both with and without wearing the EGME, as illustrated in Fig. 7B. The experimental procedure involved the following steps: participants stood upright, then performed a squat and held the squatting position for a specified duration, followed by standing up to return to a natural upright posture. The squatting and standing motions were controlled naturally by the participants and were maintained consistently throughout the repeated trials. The experiment was divided into five distinct phases: standing, squatting, squat holding, standing up, and standing.

Following the completion of the short-duration squat holding task and the thirty-minute rest period, participants were tested for the maximum duration they could maintain the squat holding position both with and without wearing the EGME. The timing began five seconds after the participants assumed the squat holding position and ended when the participants either exhibited clear signs of discomfort or voluntarily returned to an upright standing position.

## Results

### Simulation results

**Fig. 8:**
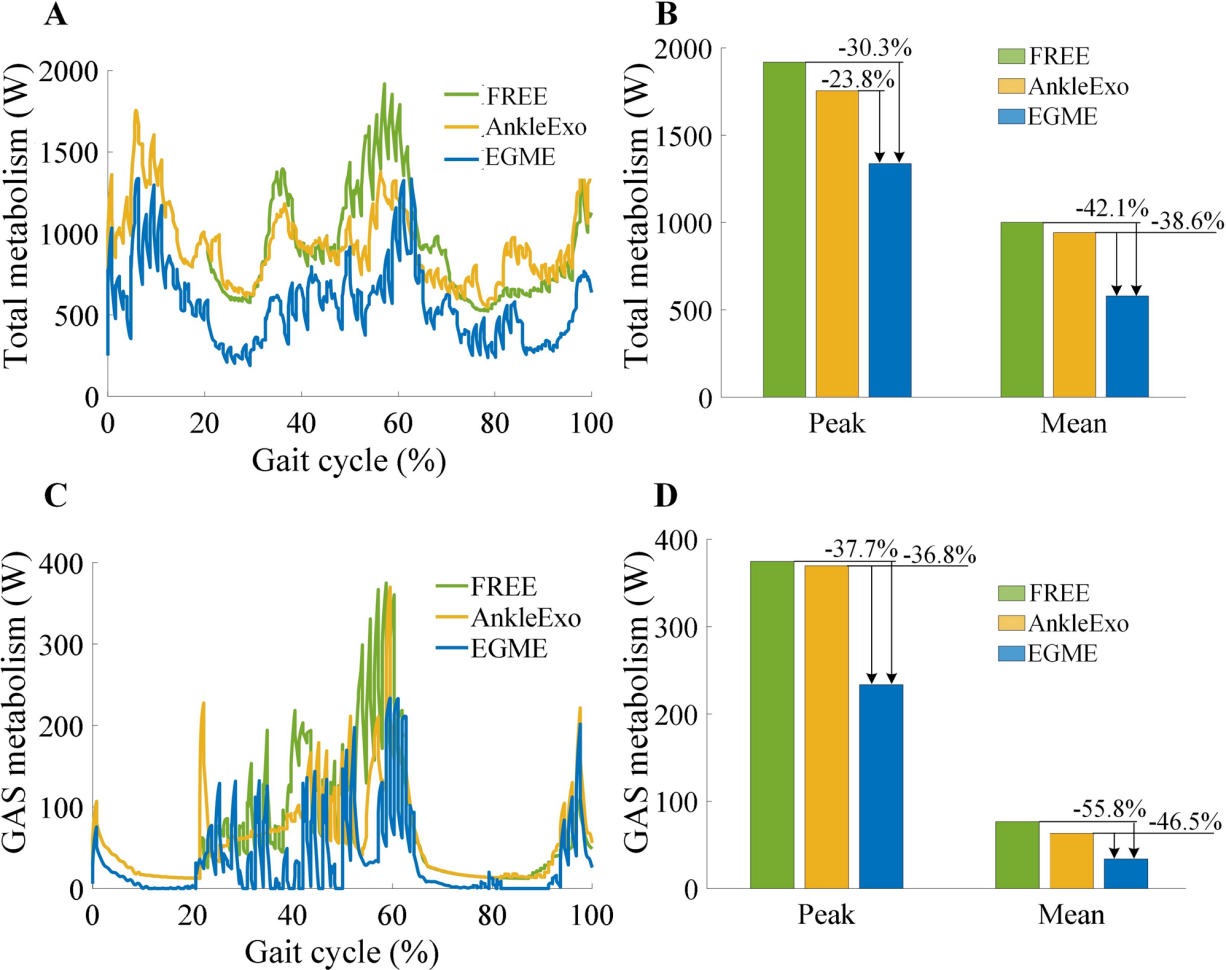
Comparison of total and GAS metabolism under FREE, ankle exoskeleton and EGME conditions. A and B are the comparison of the total metabolic curves, peak and mean values, respectively. C and D were the metabolic curves, peak and mean values of GAS, respectively

**Fig. 9:**
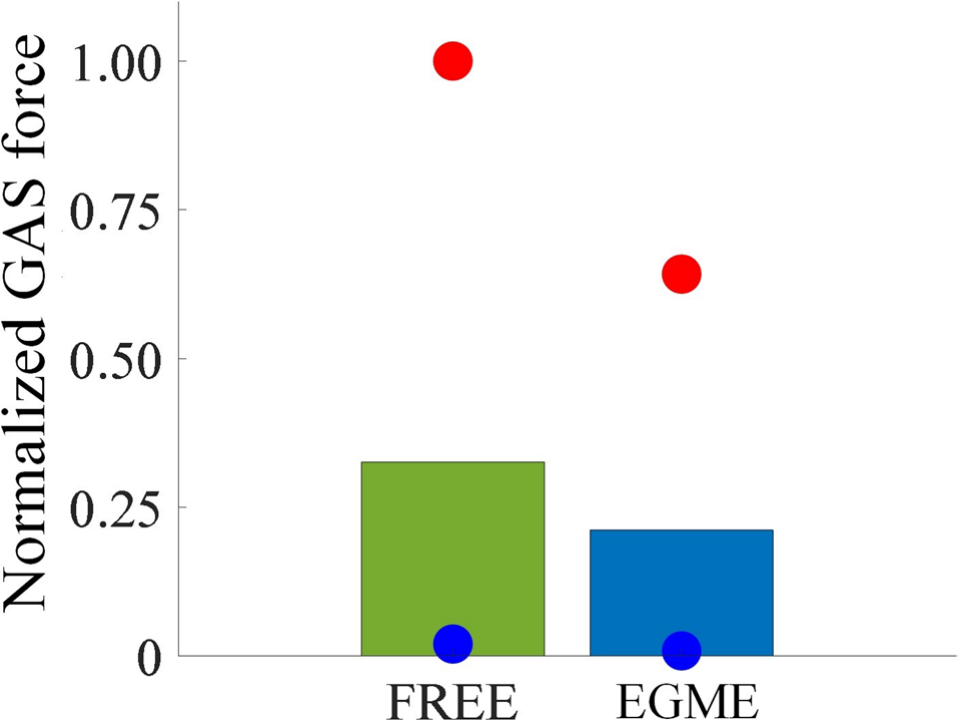
Comparison of normalized GAS force under FREE and EGME conditions. The red and blue dots represent the maxima and minima in the gait cycle, respectively

As shown in Fig. 8, the green, yellow, and blue curves represent the muscle metabolism results during the full gait cycle for the FREE (no exoskeleton), ankle exoskeleton, and EGME-assisted conditions, respectively. The metabolism simulation results indicate that, on average, the EGME-assisted condition reduces muscle metabolism by 42.1% compared to the FREE and by 38.6% compared to the ankle exoskeleton. The peak metabolism in the EGME-assisted condition is reduced by 30.3% compared to the FREE and by 23.8% compared to the ankle exoskeleton. For the GAS metabolism simulation results, the mean metabolism under the EGME-assisted condition is reduced by 55.8% compared to the FREE and by 46.5% compared to the ankle exoskeleton. The peak value for GAS metabolism is reduced by 37.7% compared to the FREE and by 36.8% compared to the ankle exoskeleton. As shown in Fig. 9, the normalized mean GAS force is reduced by 35.1% in EGME compared to FREE. This indicates that the assistance provided by EGME can reduce the output of the GAS muscle force in the human body.

### Results of the level walking experiment

**Fig. 10:**
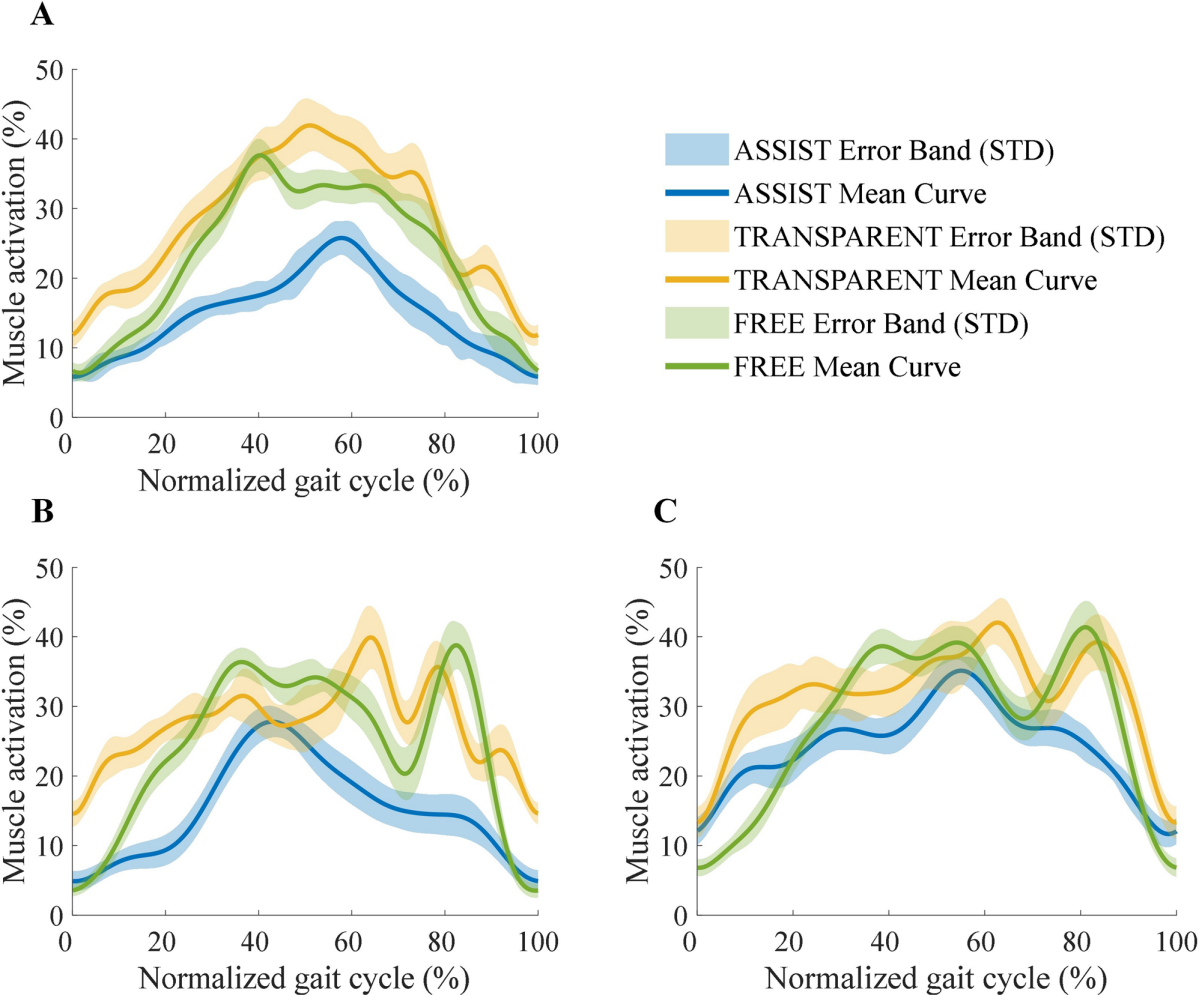
Intensity curve of GAS activation during the level walking experiment at (**A**) 1 km/h walking speed, (**B**) 2 km/h walking speed, and (**C**) 3 km/h walking speed

The activation intensity curves of the GAS during level walking are presented in Fig. 10. Subfigures (A), (B), and (C) illustrate the normalized percentage changes in the sEMG signals of the GAS at walking speeds of 1 km/h, 2 km/h, and 3 km/h, respectively, with each speed condition encompassing three different assistance modes. Specifically, the green curves represent the Free Mode (FREE) without wearing the EGME, the orange curves depict the Transparent Mode (TRANSPARENT) with the EGME set to zero-assist, and the blue curves correspond to the Assist Mode (ASSIST) with active assistance from the EGME. The shaded regions in the figures indicate the standard deviations.

**Fig. 11:**
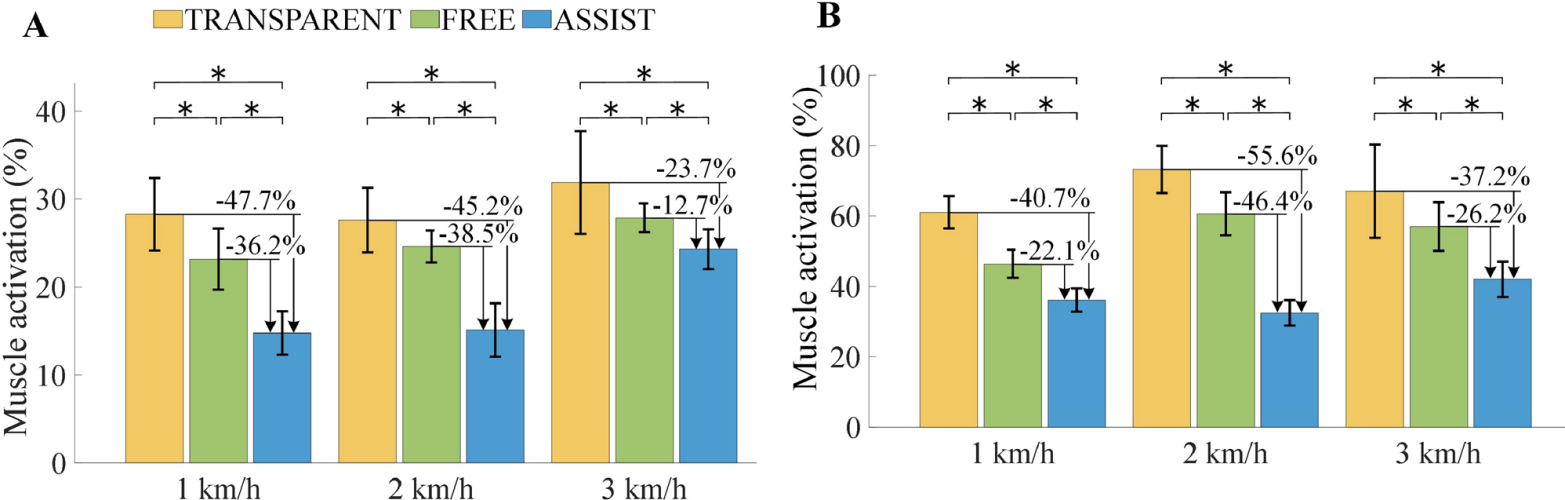
Comparison of the GAS activation intensity during the level walking experiment. (**A**) Mean activation intensity. (**B**) Peak activation intensity. The * indicates *p* < 0.05, which means that there is a significant difference between the two conditions

As illustrated in Fig. 11, the mean and peak activation levels of the GAS are shown within a single gait cycle. Subfigure (A) displays the normalized average muscle activation percentages, whereas subfigure (B) presents the normalized peak muscle activation percentages. The error bars represent the standard deviations.

At a walking speed of 1 km/h, the mean activation level of the GAS in ASSIST mode was reduced by 47.7% compared with that in TRANSPARENT mode and by 36.2% compared with that in FREE mode. The peak activation level decreased by 40.7% compared with that in TRANSPARENT mode and by 22.1% compared with that in FREE mode. At 2 km/h, the ASSIST mode resulted in a 45.2% reduction in average activation and a 55.6% reduction in peak activation compared with the TRANSPARENT mode and 38.5% and 46.4% reductions compared with the FREE mode, respectively. At 3 km/h, the ASSIST mode resulted in a 23.7% decrease in average activation, a 37.2% decrease in peak activation relative to the TRANSPARENT mode, and a 12.7% and 26.2% reduction compared with the FREE mode, respectively.

### Results of the squat task experiment

**Fig. 12:**
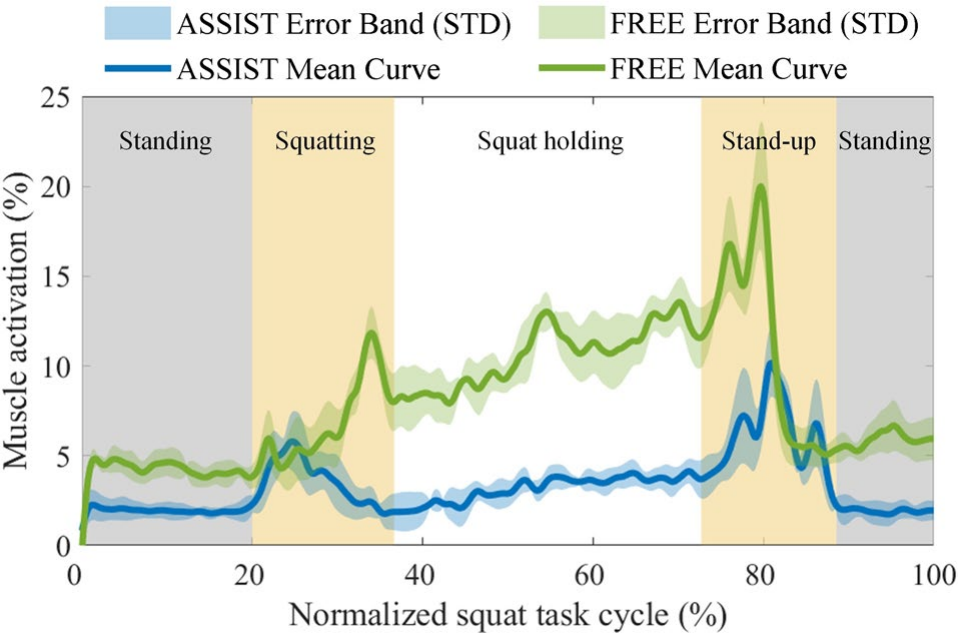
Intensity curve of GAS activation during the short-duration squat holding experiment

The results of the short-duration squat holding experiments are depicted in Fig. 12. In these graphs, the green curves represent the normalized percentage changes in GAS activation during the Free Mode (FREE) without wearing the EGME, whereas the blue curves correspond to the Assist Mode (ASSIST) with the EGME providing assistance. The shaded regions around the curves indicate the standard deviations. Additionally, the gray shaded areas represent periods when participants maintained a static standing position, the orange shaded areas denote active phases (with the left side indicating the squatting phase and the right side indicating the stand-up phase), and the white background indicates the duration of the squat holding.

**Fig. 13:**
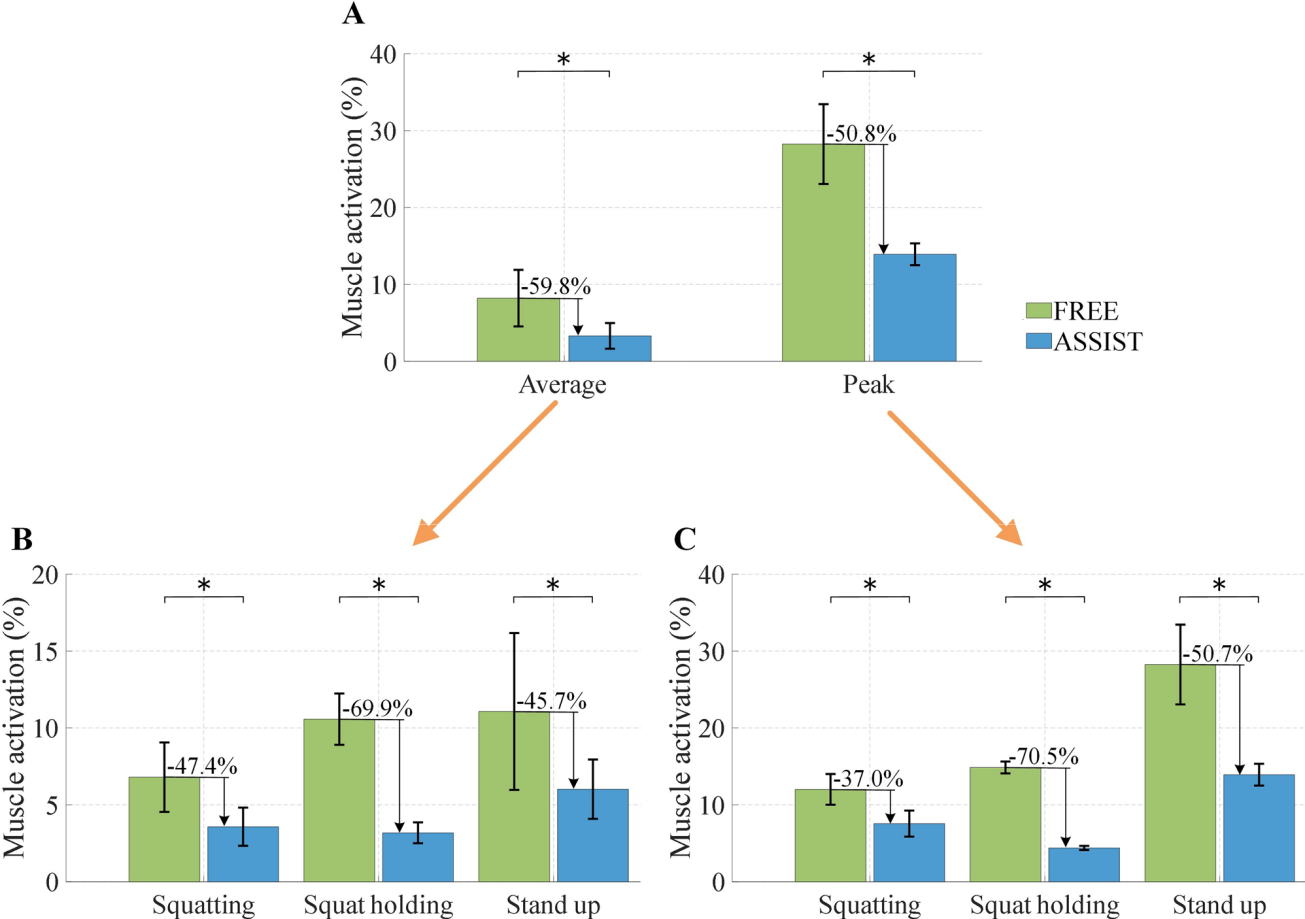
Comparison of the GAS activation intensity in FREE mode and ASSIST mode during the short-duration squat holding experiment. (**A**) Comparison of average and peak activation intensities over the full cycle. (**B**) Comparison of average activation intensity during the squatting, squat holding, and stand-up phases. (**C**) Comparison of peak activation intensity during the squatting, squat holding, and stand-up phases. The * indicates *p* < 0.05, which means that there is a significant difference between the two conditions

Figure 13 shows the average and peak activation intensities of the GAS during the entire short-duration squat holding experiment and within each phase. Figure 13A shows the normalized average and peak muscle activation percentages for the entire duration of the experiment; Fig. 13B presents the comparison of the normalized average muscle activation percentages for each phase; and Fig. 13C displays the comparison of the normalized peak muscle activation percentages for each phase. The error bars represent the standard deviations.

Throughout the entire short-duration squat holding experiment, the ASSIST mode resulted in a 59.8% reduction in the average activation level and a 50.8% reduction in the peak activation level of the GAS compared with the FREE mode without the EGME. During the squatting phase, ASSIST mode achieves a 47.4% decrease in average activation and a 37.0% decrease in peak activation relative to FREE mode. In the squat holding phase, the ASSIST mode led to a 69.9% reduction in average activation and a 70.5% reduction in peak activation compared with the FREE mode. Compared with the FREE mode, the ASSIST mode resulted in a 45.7% decrease in average activation and a 50.7% decrease in peak activation during the stand-up phase.

**Fig. 14:**
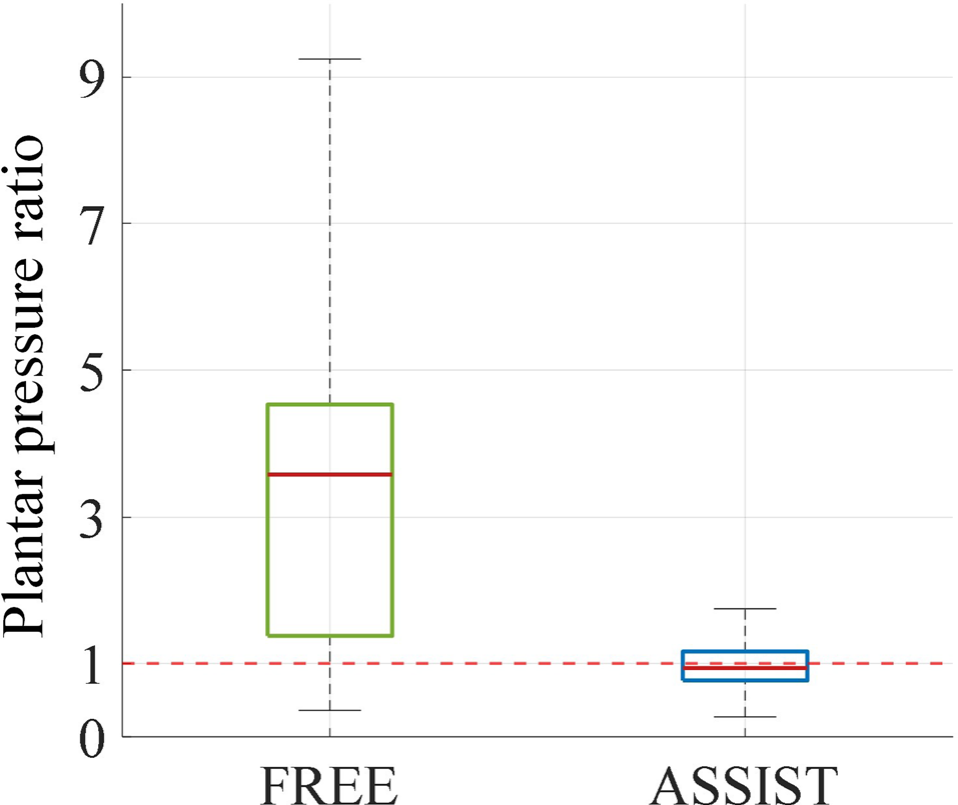
Comparison of forefoot-to-hindfoot pressure ratio distribution with and without exoskeleton

To investigate the impact of exoskeletons on ankle stability during squatting, we compared plantar pressure data between conditions with and without the exoskeleton. The boxplot shown in Fig. 14 shows the distribution of the forefoot-to-hindfoot pressure ratio under both conditions. The results indicate that, without the exoskeleton, the median forefoot-to-hindfoot pressure ratio was approximately 3.6, significantly higher than the ideal value of 1, suggesting an uneven distribution of plantar pressure. Additionally, the larger boxplot range further indicates higher variability in the pressure ratio and poorer ankle stability. In contrast, when wearing the exoskeleton, the boxplot range was significantly reduced, and the median value was approximately 0.94, close to the ideal value of 1, indicating a more uniform distribution of plantar pressure and significantly improved ankle stability. These findings demonstrate that the exoskeleton can optimize load distribution during squatting and effectively enhance ankle stability.

**Fig. 15:**
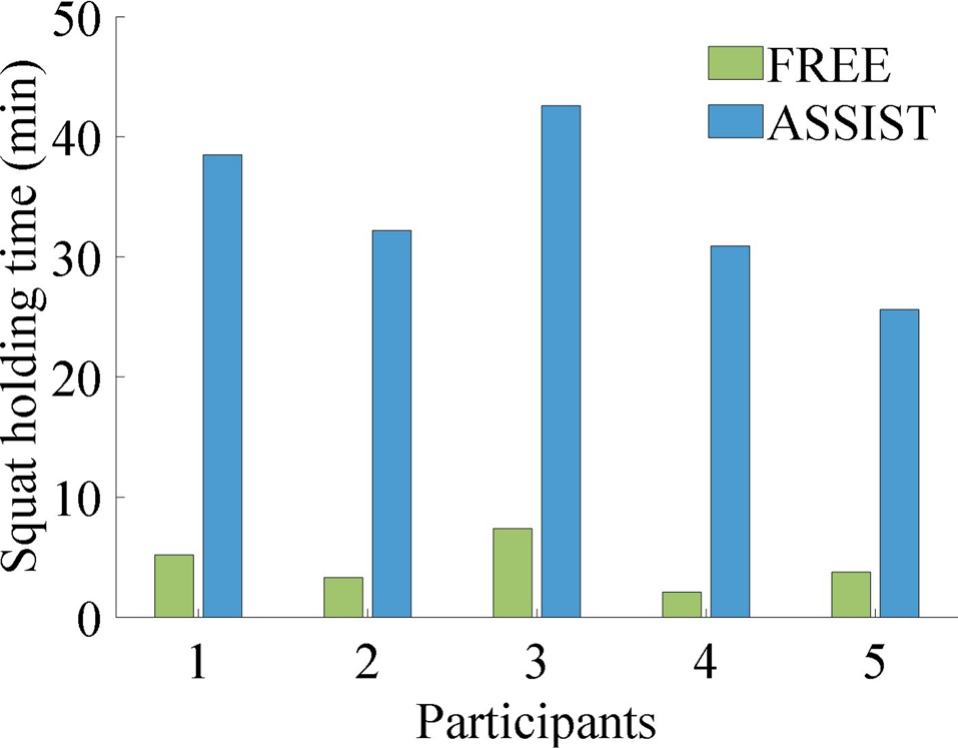
Volunteer endurance time during the long-duration squat holding experiment

As illustrated in Fig. 15, the results of the experimental investigation involving long-duration squat holding are presented. The participants who were equipped with the EGME and who received assistance were able to sustain the squat holding phase for an average duration that was 7.79 times longer than when not wearing the EGME.

## Discussion

The simulation results indicate that the proposed enhanced gastrocnemius-mimicking lower limb powered exoskeleton robot (EGME) effectively reduces the metabolic levels of the total and GAS muscles. Compared to ankle exoskeletons, it offers certain advantages in terms of reduced metabolic levels and underactuation. Experimental results show that the EGME significantly reduces GAS activation during level walking and squat tasks. In the level walking experiments, compared with the FREE mode, the ASSIST mode significantly reduced GAS activation intensity across all walking speeds. In the short-duration squat holding experiments, the average activation level of the GAS decreased by 59.8%, substantially reducing the energy output required from the human body. Furthermore, in the long-duration squat holding experiments, the participants in ASSIST mode were able to maintain the squat holding phase for an average duration that was 7.79 times longer than that in FREE mode. During the squat task, the EGME leads to a more balanced distribution of plantar pressure, reduces the fluctuations in the pressure ratio between the forefoot and hindfoot, and significantly enhances ankle joint stability. Overall, the EGME played a crucial role in supporting, facilitating forward propulsion, initiating pre-swing movements, and maintaining joint stability.

Based on EGME ‘s design characteristics and control strategy, we believe that: In the early phase of single-leg support, the EGME supports the torso and optimizes the direction of support by adjusting the force transmission angle (as governed by parameter $\:{{}^{\text{Ank}}\varvec{p}}_{\text{GA}}$ within the EGME). The direction of the applied force in the EGME is closer to the vertical, compared to the GAS. This optimization leads to a higher proportion of vertical support and reduces the effects of horizontal deceleration. In the mid-phase of single-leg support, the EGME operates in a near-isometric thrust mode, reducing the negative impact of the GAS on the forward motion of the torso. In the late single-leg support through pre-swing, the GAS continues to generate force through residual muscle activation, transferring this force to the support leg to initiate swing and forward propulsion. Due to the motor-driven characteristics of the EGME, it can overcome the continuity issues associated with muscle deactivation, providing stable forward propulsion throughout the pre-swing phase. During the squat task, due to the rigid characteristics of the EGME, it not only maintains ankle joint stability but also supports a portion of the body’s weight.

This study primarily focused on the GAS muscle, given its central role in plantarflexion and propulsion. However, lower-limb function in humans relies on the coordinated actions of multiple joints and muscle groups. Since the EGME spans both the knee and ankle joints, it may influence the coordination and kinematic coupling between these segments. Although this aspect was not addressed in the current study, future research should explore how the EGME affects the interaction between these joints. In particular, control strategies based on frameworks such as the uncontrolled manifold hypothesis (UCM) could provide insights into whether the exoskeleton preserves or disrupts natural movement synergies [50]. Furthermore, while the EGME demonstrated initial assistive effects in this study, there is still room for improvement. The current assistance may not fully meet the diverse needs of different users or movement scenarios. Therefore, future investigations should incorporate techniques such as human-in-the-loop approaches to personalize and optimize the assistance system, thereby enhancing the EGME’s adaptability and efficiency.

The EGME holds significant promise in a range of application scenarios, particularly in the context of individuals suffering from conditions related to gastrocnemius muscle injuries or weakness. One important potential application is in the rehabilitation of patients with conditions such as Achilles tendonitis, calf muscle strains, or even post-surgical recovery following lower limb surgeries. These individuals often experience difficulties with walking, such as a reduced ability to raise the heel off the ground, leading to increased tripping or stumbling risks due to insufficient toe clearance during walking. By providing targeted assistance to the gastrocnemius, the exoskeleton can help mitigate these risks, improve gait, and reduce the likelihood of falls.

It is important to note that the EGME is a proof-of-principle device, and the current experiments were conducted solely with healthy subjects. As such, the findings may not be directly applicable to individuals with muscle injuries or neurological conditions. Therefore, future studies should include clinical populations with various conditions, such as calf muscle injuries, neurological disorders, and gait abnormalities, to better evaluate the EGME ‘s potential in addressing these specific needs and improving outcomes for individuals with diverse physical conditions.

## Conclusion

In this study, we developed a biarticular exoskeleton designed to mimic and enhance the functionality of the gastrocnemius across multiple movement tasks. We analyzed and designed the EGME from both mechanical and biological perspectives. Experimental results demonstrated the potential effectiveness of the device, including reduced gastrocnemius activation during walking and squatting, as well as improved endurance in squat-holding tasks. Furthermore, the EGME contributed to more balanced plantar pressure distribution and enhanced ankle joint stability during squatting. The observed assistive effects of the EGME, which were significantly stronger than those of monoarticular exoskeletons, can be attributed to its design characteristics and control strategy, which are intended to emulate the functional phases of the gastrocnemius during gait. Specifically, the EGME provides vertical support in early stance by optimizing the force transmission angle, operates in a near-isometric thrust mode during mid-stance to reduce braking effects, and delivers continuous forward propulsion in late stance and pre-swing through motorized actuation. During squatting, its rigid structure contributes both to ankle joint stabilization and partial body weight support. In addition, the proposed structure enables the actuator to incorporate the torque requirements of both the knee and ankle joints during operation, thereby exhibiting the advantages of underactuation.

Future work could extend this approach to other biarticular muscles, improve the weight and ergonomic integration of the EGME, and evaluate its applicability in broader use cases, including rehabilitation and performance support. In addition, further investigations are warranted to assess its effects on inter-joint coordination and kinematic coupling, which could provide valuable insights for refining the design and functional control of such systems.

## Data Availability

The datasets used during the current study are available from the corresponding author on reasonable request.

## Abbreviations

EGME: Enhanced gastrocnemius-mimicking lower limb powered exoskeleton robot
GAS: Gastrocnemius
SOL: Soleus
IMUs: Inertial measurement units
sEMG: Surface electromyography
DC: Direct current
FSM: Finite state machine
DOFs: Degrees of freedom
MVC: Maximum voluntary contraction
UCM: Uncontrolled manifold hypothesis
FREE: Free mode
TRANSPARENT: Transparent mode
ASSIST: Assist mode
